# What next for the polyclinic? New models of primary health care are required in many former Soviet Union countries

**DOI:** 10.1186/s12875-022-01812-w

**Published:** 2022-08-04

**Authors:** Nigel Edwards, Igor Sheiman

**Affiliations:** 1 Nuffield Trust, 59 New Cavendish St, London, W1G 7LP UK; 2 National Research University Higher School of Economics, 0 Myasnitskaya str., 101000 Moscow, Russian Federation

**Keywords:** Primary health care, Multi-specialty model, Polyclinic

## Abstract

**Background:**

There is unfinished reform in primary care in Russia and other former Soviet Union (FSU) countries. The traditional ‘Semashko’ multi-specialty polyclinic model has been retained, while its major characteristics are increasingly questioned. The search for a new model is on a health policy agenda. It is relevant for many other countries.

**Objectives:**

In this paper, we explore the strengths and weaknesses of the multi-specialty polyclinic model currently found in Russia and other FSU countries, as well as the features of the emerging multi-disciplinary and large-scale primary care models internationally. The comparison of the two is a major research question. Health policy implications are discussed.

**Methods:**

We use data from two physicians’ surveys and recent literature to identify the characteristics of multi-specialty polyclinics, indicators of their performance and the evaluation in the specific country context. The review of the literature is used to describe new primary care models internationally.

**Results:**

The Semashko polyclinic model has lost some of its original strengths due to the excessive specialization of service delivery. We demonstrate the strengths of extended practices in Western countries and conclude that FSU countries should “leapfrog” the phase of developing solo practices and build a multi-disciplinary model similar to the extended practices model in Europe. The latter may act as a ‘golden mean’ between the administrative dominance of the polyclinic model and the limited capacity of solo practices. The new model requires a separation of primary care and outpatient specialty care, with the transformation of polyclinics into centers of outpatient diagnostic and specialty services that become part of hospital services while working closely with primary care.

**Conclusion:**

The comprehensiveness of care in a big setting and potential economies of scale, which are major strengths of the polyclinic model, should be retained in the provision of specialty care rather than primary care. Internationally, there are lessons about the risks associated with models based on narrow specialization in caring for patients who increasingly have multiple conditions.

**Supplementary Information:**

The online version contains supplementary material available at 10.1186/s12875-022-01812-w.

## Highlights

• The Semashko polyclinic model has lost strengths due to excessive specialization.

• Solo and group PHC practices are no longer suitable to manage multimorbidity.

• A new ‘extended general practice’ reorients the health system towards PHC.

• Restructuring polyclinics is possible by transforming them into outpatient specialty units of hospital structures.

**Supplementary Information:**

The online version contains supplementary material available at 10.1186/s12875-022-01812-w.

## Background

In the former Soviet Union (FSU) and some Central and Eastern European (CEE) countries, the traditional ‘Semashko’ Soviet multi-specialty polyclinic model, originally developed in the 1930s, has been retained [1]. However, in many rural and some urban areas of FSU countries, the traditional polyclinic model shifted towards solo and group primary care practices in the 1990s in response to policymakers’ demands for stronger primary health care (PHC). This shift towards solo and group practices continues today in many countries of this region [2].

The existing PHC models are currently facing a number of challenges. The professionals running standalone practices are struggling to respond to a growing proportion of people with multi-morbidity and complex healthcare needs, in a context of underdeveloped and underfunded supportive services, such as social care, rehabilitation, long-term care and palliative care [3]. While in theory, the mostly urban polyclinic-based generalists could be delivering more comprehensive care, in practice, patients seek out ‘narrow’ specialists based in polyclinics (cardiologists, neurologists, etc., further referred as specialists) instead for the management of relatively common conditions that would be in the scope of PHC providers in other countries [1]. Neither the polyclinic approach taken in urban areas nor the rural solo/group practice are functioning well and new approaches are needed.

In this paper, we focus on the Russian Federation and consider a number of questions that policy makers, payers and medical leaders could discuss when planning changes to their services. Firstly, what are the current strengths and weaknesses of the polyclinic model currently found in Russia and other FSU countries and does this mean that there are some aspects that should be preserved. Secondly, what are the features of the emerging multi-disciplinary and large-scale primary care models internationally. Thirdly, which elements of the new models of primary care could be adapted to the Russian and other similar settings. We argue the case that polyclinics should be transformed – not into the model of standalone or small group practices that is common – but instead into the ‘extended general practice’ model seen across Europe that re-orients the health system towards comprehensive PHC delivered by multi-disciplinary teams.

## Methods

The evidence on the strengths and weaknesses of the polyclinic model set out in this paper is based on a review of the literature and two physician surveys. The review is focused on: a) determining characteristics of multi-specialty polyclinics in Russia, indicators of their performance and the evaluation in the specific country context; b) description of the emerging extended PHC practices internationally; c) comparison of the two models. We searched MEDLINE using the query: (Ambulatory Care Facilities[mh] OR polyclinic) AND (model OR type OR semashko) AND (USSR OR russia OR europe OR european union) AND 1990:2022[dp]. 1614 findings were checked manually and 36 were relevant. We also used sources snowballed from these reports and the grey literature related to Russian health care, including those in limited circulation, unpublished documents, memorandums, and presentations from our personal collections covering more than twenty years.

The surveys of Russian physicians are designed to explore the managerial environment for their performance of the staff in polyclinics. Firstly, the managerial control is evaluated via questions like: Is the number of patient visits planned by the polyclinics’ administrators? Does the failure to implement plans can cause a reduction in physicians’ remuneration? Who determines the average length of a patient visit and what happens if it is regularly violated? Are physicians involved in managerial actions to improve the performance of polyclinics?

Secondly, we assess the level of physicians’ clinical autonomy. The examples of questions: Do you select patients for check-ups and screening or rely on administrative decisions? Do your referrals to hospital admissions and CT/MRI tests require the authorization of polyclinics administrators? Which indicators of performance are used for your reporting to the administration?

Thirdly, teamwork and coordination of providers in polyclinics is evaluated. We ask questions about their joint planning of curative activities, inter-discipline consultations and training sessions, as well as the leading role of district physicians in the team—in joint planning and managing chronic cases.

International comparisons of primary care performance are based on the national and OECD databases.

### Survey 1

A small-scale survey of polyclinics’ physicians was conducted in January 2020 in three urban polyclinics in Moscow city and Moscow oblast (the region near Moscow). They represent a big multi-specialty urban polyclinic with an average number of staff of around 80–90 health workers. In Moscow city, these polyclinics have been consolidated into bigger outpatient centers with three to five polyclinics each. But this new level of administration was not taken into account, to reflect the usual pattern of administration of polyclinics in Russian big cities. The questions relate only to the staff of individual polyclinics (not their amalgamation). The special area of interest is the attitude of generalists and specialists. A list of 13 questions (appendix 1) was sent to all three polyclinics’ physicians through the Russian social network “Vkontakte”. This survey was anonymous, respondents were not compensated and were reassured that any negative feedback would not affect them. The postgraduate students of the National Research University Higher School of Economics (Moscow) dispersed the survey. It was sent to 129 physicians, 103 physicians (80%) responded, including 67 district physicians and 37 specialists. The questionnaire had the same questions to all respondents, and the latter were required to answer all questions by the design of the survey. Therefore, the response rate was the same for all questions. Similarly, the fraction of participants and respondents was the same for each question. Ten physicians on the list were randomly selected and approached directly for face-to-face interviews.

This small-scale survey doesn’t represent all physicians in the country, but it can provide additional evidence to our observations on the limitations of professional autonomy in polyclinics. Some questions from this survey were also used in our recent study of the national preventive program [4].

### Survey 2

This survey was designed to receive more detailed evidence of the level of interaction between professionals in polyclinics, including the exchange of information about patients’ emergency calls, the level of awareness of patients’ hospital admissions, the involvement of polyclinics' physicians in the rehabilitation activities after hospital admissions of stroke and heart attack cases. A special area of interest is the referral pattern of district physicians: what is the share of first visits that is referred to specialists?

The second survey was conducted online in October 2020 in the middle of the COVID-19 pandemic through the mobile ap “Handbook of Physician” (available in Google Play and AppStore) with 540 thousand registered users. 2316 physicians responded to the survey. They represented 81 of 85 regions of the country. 1118 respondents worked in polyclinics (48%), 1068 – in hospitals (46%), the rest – in other settings. Since the survey was designed to look at broader issues of service integration (between polyclinics, hospitals, emergency care centers, etc.), we selected respondents that worked in polyclinics and studied their responses to the questions about the level of integration *within* polyclinics. All questions were asked with the note “in regular conditions of work before March 2020”. This part of the questionnaire is provided in appendix 2.

Survey 2 covers generalists and specialists in the staff of polyclinics in practically all types of primary care settings in Russia which vary in size. A high popularity of the ap in all Russian regions and a substantial number of respondents that represent various medical organizations and physician specialties make the survey a reliable instrument of the study.

## Results

### The polyclinic model

The polyclinic model was established in the USSR in the early 1930s and inherited by FSU and some CEE countries. The polyclinic is a multi-specialty entity providing both primary care and most outpatient specialty care. Typically, there are separate clinics for adults and children and each has a catchment area and a patient list managed mostly by district therapists, district pediatricians and general practitioners (GPs) – all of which are collectively referred to as ‘district physicians’ (DPs). Mental health care is not provided by this service – this is the area of specialty organizations. GPs with a broad task profile are only emerging and account for only 15% of DPs. The catchment population of polyclinics in big urban areas ranges from 30,000 to 120,000 people [5].

People can choose a polyclinic, and most choose the provider closest to their place of residence. Patients enrolled in a polyclinic form the patient list. According to federal regulations, DPs and GPs act as gatekeepers and refer patients to specialists and hospitals. But many regions loosen the requirements of gatekeeping: patients increasingly see specialists directly without a referral from primary-care physicians.

There has been a trend towards specialization within PHC since the 1990s. The Semashko model of a district unit with mainly a district physician has given way to multi-specialist polyclinics, which currently employ 15–20 categories of specialists in big urban areas (including for example, cardiology, gynecology, surgery, etc.), and three to five categories in small cities. Rural and small city areas are served mostly by small solo practices. Outpatient specialists account for around two-thirds of polyclinic staff and service activity [5]. It is important to note that the increased professional diversity of the polyclinic staff has been based on a growing number of medical specialists rather than nurses and other allied health professionals. The role of nurses is limited to non-clinical functions. The nurses to physicians ratio is only 2.1, as against 3.0 in Germany, 3.8 in Canada and 4.3 in the US ([6], p.179).

Legislation defines the polyclinic as a PHC model, which consists of ‘primary physician service’ (care provided by DPs) and ‘specialty primary care’. The latter includes some care equivalent to that provided by outpatient specialists in Western countries, but also care that is effectively managed by family doctors elsewhere, for example, angina and type 2 diabetes.

The governance of polyclinics is highly centralized. The regional health authority appoints directors of polyclinics and manages their performance, most of the rules and patterns of care provision are set by the federal Ministry of Health. The administration of polyclinics is a multilayered structure: a director, a medical director, a few deputies, the head of the district unit, and heads of specialty and diagnostic units. Most polyclinics are state owned and staffed with salaried medical personnel. The growing private sector is also based on this model.

The majority of a polyclinic’s financial revenue is derived from a regional mandatory health insurance (MHI) fund on a capitation basis. But the revenue of a polyclinic can be reduced if it has not reached a minimum number of visits. This target is set and controlled by the regional health authority and MHI fund (which jointly act as a purchaser of care). When there is a risk of not reaching a planned number of visits, the administration of polyclinics has to encourage multiple referrals of patients. Polyclinics’ preventive services are paid for on a fee-for-service basis. The rates of payment are set for a fixed package of services under a so called “program of dispensarization” (a nation-wide vertical program of check-ups and screenings). The control of the actual number of preventive services is conducted by MHI funds and administration of polyclinics. While this method of payment motivates physicians to implement the program, it limits their professional autonomy on the choice of preventive services for an individual. They have to provide the entire bundle of services to be reimbursed, irrespective of the actual need of a patient [4].

The salary of medical personnel has fixed and variable parts. The latter is based on some pay-for-performance indicators, including the number of visits managed by the individual physician, number of patient complaints and some preventive services. The variable part is determined individually by administration.

### Evaluation of the model

There is an unfavourable context for the operation of the urban polyclinic model in modern Russia. There are low levels of health funding (currently, public funding is around 3.5% of GDP), there is a 30% shortage of district therapists, structural distortions in the workforce which are explored below, a hospital-centred model and little focus on chronic disease management [7].

A major strength of the model is its capacity to provide an easy access to primary and specialist care, at least in theory. Patients can see a DP, receive diagnostic services and have consultations with specialists ‘under one roof’. Specialists may or may not be located in the same premises. But even if they are, this does not mean that patients can have tests and see specialists the same day. This process usually takes weeks because of the shortage of specialists [7].

Polyclinics have a number of potential advantages due to the consolidation of service delivery. These include additional leverage to implement care pathways and shared use of capital investment resources. Polyclinics can also centralize administrative and support services, with potential economies of scale. Furthermore, large settings can redistribute resources across geographic areas through setting up small branches in remote areas and polyclinic workers can stand in for each other in case of illness or holidays. There is evidence that these strengths are not fully realized in Russia and managerial action is needed to deal with this [8].

Another potential strength of the polyclinic model is better financial sustainability relative to solo and group practices. The model has enabled the introduction of a fundholding scheme in some regions of Russia, with polyclinics as major financial risk-bearers. Within a short period of its implementation (four to five years in most regions), this has allowed strong economic incentives to be used to increase the role of PHC in the health system [5].

The larger scale of polyclinics also allows them to respond to national health programs more effectively. For example, larger scale has allowed the implementation of the recent federal program called “Resource-saving polyclinics” that covers most big polyclinics in the country. The objective is to improve the efficiency of internal processes through new appointment systems, separation of patient flows across individual providers, improve electronic communication and develop better organization of the working space, etc. These innovations are more cost effective in big settings. The response to COVID-19 is also facilitated by large-scale facilities.

There are, however, a number of major weaknesses of the polyclinic model.

First, *strong administrative pressure on physicians in polyclinics and their limited professional autonomy*. Physicians are poorly involved in the management of polyclinics. They work according to the rules determined by polyclinics’ administrators, who in turn follow the commands of the federal and regional health authorities. Administrators set DPs’ catchment areas, plan the number of patient visits, develop the norms for the average length of a patient visit, determine the scope of preventive services and their coverage, ration expensive diagnostic resources for each physician and approve referrals to hospital.

The first survey provided evidence of managerial control and limited professional autonomy:

- 66% of physicians reported that plans for the number of patient visits are developed by the polyclinics’ administrators. Only 34% plan this activity themselves.
- 59% indicated that the failure to implement plans on the number of visits can cause a reduction in their remuneration.
- 66% reported ‘administrative action’ if the norms for the average length of a patient visit are regularly violated.
- Only 25% of physicians select patients for check-ups and screening programmes themselves after assessing their risk factors. The rest rely on administrative decisions on the targeted populations. The share of those who select patients for chronic disease management themselves is higher though – 69%.
- Just under 50% reported that their referral of patients to hospital requires the authorization of heads of specialty units, 15% – the deputy director, 12% – medical commissioners and 6% – other actors. There is a similar distribution of responses for the authorization of CT and MRI referrals.
- 28% of respondents reported administrators’ ‘excessive administrative regulation’ of clinical decisions.

Second, *the loss of the primary care providers’ leading curative and coordination roles*. In Russia, DPs are traditionally seen as gatekeepers for access to specialty services. Theoretically, they are supposed to act as a patient’s guide through the health system and ensure continuity of treatment. However, a high level of polyclinic care specialization distorts the coordination role of primary care providers. In a big multi-specialist entity with a growing division of labour and many specialists working together, many traditional curative functions of DPs are delegated to specialists. It is hard for DP to resist the temptation to refer a patient to a specialist next door. Clinical recommendations, pathways and quality control actors tend to incentivise specialist consultation. Polyclinics’ administrators often encourage referrals to specialists to meet the specified minimum number of visits. The task profile of DPs’ curative work therefore gradually narrows. They deal with a few simple diseases, and the majority of patient care (even gastritis, ulcer, bronchitis) is managed by specialists [5]. Contrary to GPs, district physicians are allowed to practice without postgraduate training. As a result, a DP’s coordination role also narrows.

The first survey provides the following evidence of a lack of cooperation and coordination in the urban polyclinic:

- 35.5% of DPs said that the development of plans for the joint management of cases by DPs and specialists did not occur, 45.2% said it ‘rarely’ occurred, 9.7% said it ‘sometimes’ occurred and only 1.6% said it ‘always’ happened. This is contrary to the expectation that a big entity facilitates joint working.
- Training sessions for DPs with the involvement of specialists were reported as a regular event by only 4% of polyclinics’ physicians, as a rare event by 35%, while the rest of the respondents indicated their absence.
- Only 3.2% of respondents reported medical case conferences as a regular event.

This survey allows us to determine a task profile of DPs and their role in a multi-specialty team:

Only 29,7% of DPs reported that they referred to specialists less than 10% of patients, that is had referral patterns similar to European GPs who referred from 5 to 15% of patients to specialists [2]. The bulk of Russian physicians referred every second patient to specialists, indicating an excessive specialization of primary care and a limited task profile of generalists. Unsurprisingly, only 26% of DPs reported themselves as “captains of the team” in joint planning of curative activities.

The second survey provides a more detailed evidence of a level of integration in Russian polyclinics. Its results were compared with similar estimates made in 2012 [9].

**Fig. 1:**
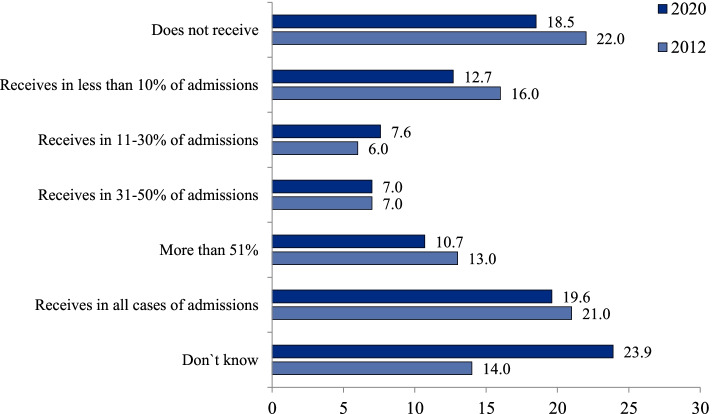
Distribution of polyclinic physicians’ responses to the question `How often does your polyclinic receive information about hospital admissions of patients in the catchment area`, % (survey 2)

**Fig. 2:**
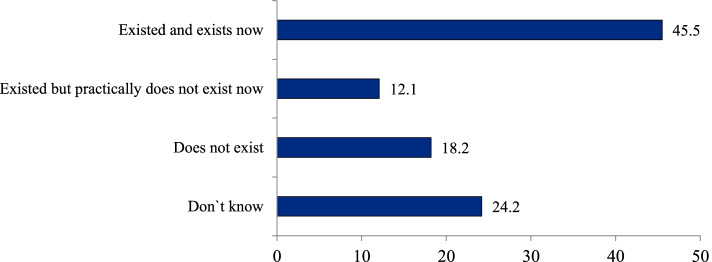
Distribution of polyclinic physicians’ responses to the question `Does your polyclinic have a regular practice of visiting patients within first days after their hospital admission with a stroke or a myocardial infarction`, % (survey 2)

An important indicator of the interaction of polyclinics and hospitals is the level of information exchange between them. Only 19.6% of respondents said that their polyclinics received the information about all hospital admissions of patients in their catchment area in 2020; 18.5%—didn’t receive it at all; 23.9% could not answer this question, which was close to the negative answer. The level of physicians’ awareness of hospital admissions in 2020 was even less than in 2012 (Fig. 1). This in turn complicates the continuity of care after hospitals admissions. The survey indicates that even for “catastrophic” cases of a stroke or a myocardial infarction the practice of visiting patients within the first days of their hospital admission is not common: it is reported only by 45.5% of respondents (Fig. 2).

Substantial efforts have been undertaken recently in introducing modern IT in medical facilities, but a national electronic medical record system has not been built yet. Only a few big cities have a system that covers both outpatient and inpatient facilities. The majority of outpatient physicians don’t know much about care utilization on other stages of service delivery.

Third, *the limitation of outpatient specialists’ task profile and curative competences.* For the reasons mentioned above, a multi-specialist polyclinic generates demand for specialty care*.* This demand is served by two specific types of specialist who provide only outpatient care: those who work in polyclinics dealing only with simple cases as first-contact providers; and those who provide only inpatient care for complex cases. The former face the problem of professional isolation from their counterparts in hospitals and have limited clinical competences, for example, they do not carry out operations or manage difficult cases. The latter have very limited responsibilities for outpatient consultations. Thus, a multi-specialist polyclinic not only generates demand but also requires a growing supply of specialists. Only 13% of physicians in Russia are generalists [5], compared with 27% in the UK, 29% in France and 48% in Canada, with 23% being the average for the OECD countries [6].

Fourth, *the lack of economic incentives in a multi-specialty polyclinic*. A salaried status and the principle of a ‘common pot’ inherent in a big entity decrease the economic motivation of polyclinics’ health workers relative to their self-employed counterparts in solo and group practices. The first survey of physicians indicates that their income is poorly linked to the financial revenue of the polyclinic: 49% of respondents reported that they did not see this link, 37% saw the link, while the rest did not answer.

Fifth, a *low potential for patient choice of PHC providers and their competition*. Russian citizens have a strong interest in provider choice but the majority cannot choose due to the prevalence of big entities. Also, there is a growing interest in physician practices that are smaller and therefore closer to patients’ homes.

### Some indicators of polyclinics’ performance

Large-scale provision of primary care in polyclinics might be expected to reduce the burden on hospitals, but this has not been the case. In spite of a relatively high number of outpatient physician consultations (9.9 per person), the hospital admission rate in Russia is 52% higher than the OECD average and even higher than in European countries, including Estonia, which had similar high levels of admission in the first post-Soviet period and then had rejected a multi-specialty polyclinic model. A poor-performing primary care system in Russia increases the probability of acute deterioration in people living with asthma, chronic obstructive pulmonary disease, congestive heart failure and some other illnesses, therefore requires hospital admissions that are avoided internationally. Together with a high average length of hospital stay, a significant frequency of admissions produce a very high total utilization of inpatient care: bed-days per capita in Russia is more than two times higher than the OECD average (Table 1). Similarly, the utilization of emergency care per capita in Russia is nearly three times higher than the average for OECD countries [5].

**Table 1:** Some indicators of health care utilization in Russia, selected European countries and OECD average in 2019 or nearest year

	Russia	UK	France	Germany	Italy	Estonia	OECD average
In-person doctor consultations per person	9.9	-	5.9	9.8	10.4	5.5	6.8
Hospital discharge rates per 1000 population	222	127	184	252	113	125	146
Average length of stay in hospital (days)	10.6	6.9	8.8	8.9	8.0	7.7	7.6
Number of bed days per capita^a^	2.4	0.9	1.6	2.2	0.9	1.0	1.1

^a^Authors’ estimate based on discharge rates and ALOS

Source: [10]

This can be attributed to a number of factors. Firstly, inpatient care remains a major priority of health policy, with a major proportion of funding going to this sector. Secondly, the curative capacity of outpatient specialists in polyclinics is lower than that of their counterparts in hospitals because they deal only with relatively simple cases. Thirdly, patients prefer to be admitted to a hospital due to a traditional mistrust of polyclinic physicians. Administrative pressure – a major characteristic of the model – does not allow patients’ trust to increase.

A theoretically important feature of the polyclinic model is its focus on preventive activities but this does not happen in practice. Physicians and other professionals have no discretion over their patients’ involvement in large-scale health programs and cannot adapt them to the specific environment of their work. An example is the current federal program of ‘dispensarization’, which covers all adults with check-ups and screenings and is implemented according to standard rules. Polyclinics’ physicians are not involved in the design of the program and cannot choose the scope of preventive activities, the targeted populations or the forms of follow-up activities for identified cases of chronic disease. This has caused a number of serious problems: an overburden on DPs, distortions in reporting, poor communication between providers of preventive and curative services, excessive prescribing of diagnostic tests and a lack of follow-up activities for identified cases. The survey of physicians conducted online in April 2019 (randomly selected 1103 physicians) demonstrated that only 7.7% of respondents indicated that a set of actual curative activities met the requirements of a pattern of dispensary surveillance issued by the Federal Ministry of Health. The analysis of medical records of 7043 patients after their hospital admissions with a stroke or a myocardial infarction indicates that nearly half of these patients have not seen a doctor during the year prior to admission [11].

Heart attacks and other cardiac ischemia mortality rate in Russia is 310 per 100 000 population (in 2019) – nearly three time higher than the average OECD (110). There is a similar gap is for stroke mortality (180 vs 60) ([10] p. 91).

Polyclinics’ responses to challenges resulting from the COVID-19 pandemic demonstrate both the strengths and weaknesses of the model. There is some evidence of its potential for the mobilization of resources, which has allowed polyclinics to allocate resources to the most vulnerable areas of service delivery and organize flexible testing and tracing of patients and their contacts. This has been facilitated by polyclinics being instructed to implement government policy through decrees and commands. On the other hand, physicians in polyclinics do not have the competences required to take on a major role in triaging or managing new cases. Hospitals have become overburdened as they have taken on this role. The excessive specialization of primary care has also prevented continuity of outpatient care for people with coronavirus.

## Discussion: where next for primary care in FSU countries?

### Developing a new approach – learning from elsewhere

FSU and some CEE countries are seeking a new model of PHC that combines the strengths of solo, close-to-home practices and large multi-specialist polyclinics while addressing their weaknesses. A shift to the model of small independent primary care practices, that have been common in Western Europe, may not be a reasonable alternative to the polyclinic model. Firstly, because patients in Russia do not favour this model and its historic legacy means they will try to bypass it. Secondly, the scale of change in the workforce required would be very large and potentially impractical. Thirdly, and most significantly, it would potentially mean losing the opportunity to adopt a more modern and appropriate approach to primary care rather than copying an old model that is starting to exhibit difficulties.

In a number of countries there has been growing interest in the development of larger multidisciplinary primary care practices or networks in response to the growing complexity of patients, the desire to provide more care locally, demand for extended hours and pressures on the workforce. In Spain [12], France [13] and the UK [14] it is increasingly common for primary care services to cover in excess of 20,000 population and these are very different from the existing polyclinics as they rely on a much wider spectrum of primary care expertise including pharmacists, a number of different therapy disciplines, mental health professionals, dentists, opticians, hearing aid technicians and dieticians. They also have an extensive role for nurses. They may also provide a base for social work and staff who can assist patients with non-medical problems and who can direct people to services that can help them. Larger units may have administrative staff to reduce the time taken on non-clinical tasks by health professionals.

The main points of difference to the current multi-specialty polyclinic model in Russia are the following:

- The level of narrow specialization of care is much higher in the polyclinic model than in an ‘extended general practice’ model. Groenengen et al. (2015) [15] found that the median number of additional professionals in extended general practice is five to six in Australia, England, Iceland, New Zealand, Poland, Slovenia, Spain and Sweden, while in Russia it may reach 20 categories [5]. This excessive specialization has destroyed the polyclinic’s original design as a centre of PHC based on teamwork, coordination and continuity of care and resulted in a fragmented provision of services with the duplication of specialists in outpatient and inpatient settings.
- The curative and coordination role of generalists remains central in the extended practices in Western Europe, while it tends to be small in the polyclinic model: specialists replace rather than supplement generalists.
- Polyclinics have extended their scope through the introduction of new categories of outpatient specialists. In contrast, while some new primary care models may include some specialists, they rely on GPs with a wide range of skills, including the ability to manage many conditions.
- Paediatric and adult care are generally provided under the same roof in the new extended primary care models, rather than in separate clinics as is often the case in urban areas in FSU countries.
- Clinicians in the new extended primary care practices are expected to follow guidelines and are subject to quality audit, but generally have more autonomy in decision-making than doctors in polyclinics.

### Developing multi-disciplinary group practices

With some considerable work, the existing polyclinics could be transformed into the type of multidisciplinary group practices described above. These would be linked to smaller satellite primary care centres to enable easy access for patients. These networks should take responsibility for the primary care of the whole population – including children – and where they are in separate facilities they should be brought together. A minimum population of 20–30,000 people will provide a critical mass to allow a wide range of primary care services, as around 10–15 GPs working together would justify hiring other professionals, including nurses and pharmacists. Larger networks, which are easier to create in urban areas, could allow even more services to be developed, including diagnostic imaging and a base for visiting specialists.

At the core of the ‘extended general practice’ model are GPs. District therapists and some of the narrow specialists would need extensive retraining to undertake the role of GP. In those systems that have retained paediatricians, it may be better to improve their capability as clinicians for children through enhanced training and to integrate them into the primary care centres, working alongside family doctors. Such a change may be easier than trying to retrain doctors who have had a purely paediatric training to work in family medicine (and vice versa) – not least because in many FSU countries, sub-specialisation in paediatrics takes place very early in training. This is also likely to be more acceptable to the population, who are used to taking children to see paediatricians, and there is some evidence that specialist primary care for children produces better outcomes [16, 17]. Locating GPs and paediatricians in the same practice would also allow issues relating to the whole family context to be understood.

Nurses in the multidisciplinary group practices would need to deliver many more services than the quasi-administrative roles that many currently undertake. There would need to be a significant programme of training for them and the wider range of primary care professionals that the model requires (mental health workers, social workers, physiotherapists etc.). Changes in regulatory rules will be required in a number of countries to allow for this, in particular to permit nurse prescribing.

The existing model, in which a lot of activity has been generated by annual screening, pre-employment checks, the issuing of sick certificates and referral to other providers, would need to be replaced. A more proactive approach based on risk assessment and the selection of preventive services for each individual patient would also be required. The new model would also necessitate a significant change in the mindset of staff and patients.

The long-term goal for the extended general practice model is that it will encompass the broad range of primary care services including rapid access for immediate problems and the management of many long-term conditions. Elements of women’s health and less serious mental health conditions would be part of this. For long-term conditions the role of specialists would be to provide assistance with diagnosis, develop management plans, provide advice where changes in these plans are needed, support or take over the management of very complex or refractory cases and, where required, periodically review the care plans and the patient’s progress. To do this means that specialists will see fewer patients and these will be more complex. They will also need to be available by phone or email to provide advice and there will be more emphasis on support, education and training provided to primary care clinicians. The reimbursement system will need to recognise this and, in those countries where it is an issue, also consider the income lost to doctors from informal payments.

The goal of integrated care would take a long time to realize, which requires a set of integrative activities in both multidisciplinary group practices and hospitals. These include:

- expanding capacity for direct information exchange
- joint planning of care through the chain of providers
- establishing process and outcome indicators for chronic disease management
- increasing people’s economic motivation for integration
- restoring GPs’ gatekeeping function and coordination role

Most importantly, regulation is needed to promote a greater interaction of GPs with specialists and hospitals beyond a simple referral system. This could include the provision of email and telephone advice, education and training, clinical supervision and other joint activities.

Specialists who currently work in polyclinics would need to be retrained either to work in primary care or to bring their skills up to a level that is appropriate for hospital outpatient/ inpatient services.

The preferable option for outpatient centres is to employ specialists who work in both inpatient and outpatient settings. To facilitate this process, the centres should become structural units of hospitals. This would avoid specialists’ professional isolation and may decrease demand for the number of specialists in the entire health system.

In most cases, access to specialist opinion would be provided by hospital outpatients departments and would be by referral from a primary care doctor (and over time by other clinicians). This would need to be supported by the development of guidelines and pathways. To be most effective, primary care would need to have access to a range of laboratory and imaging tests to help improve the appropriateness of referrals.

One area where more rapid progress could be made is in the development of primary care and outpatient consultations by digital means. Health systems have been rapidly adopting such approaches in response to the COVID-19 pandemic, although there were already a number of examples before the crisis where substantial amounts of care were delivered through these means. In common with many other countries, rural areas have great difficulty recruiting medical and nursing staff but access to smartphones with internet capability is growing rapidly and, subject to there being reasonable mobile phone coverage, a combination of digital and mobile services could help to fill this gap.

## Conclusion

FSU countries should ‘leapfrog over’ the phase of developing solo practices and build a multidisciplinary model similar to the extended general practice model seen across much of Europe. The latter may act as a ‘golden middle point’ between the excessive specialization and administrative dominance of the polyclinic model and the limited capacity of solo practices. The new model requires a separation of primary care and outpatient specialty care, with the transformation of polyclinics into centres of outpatient diagnostic and specialty services that become structural units of hospitals. A transition to this model requires retraining specialists, extending the task profile of traditional district physicians, therapists and paediatricians, consolidation of adult and child primary care, and increasing the role of nurses and other professionals. The comprehensiveness of care in a big setting and potential economies of scale, which are major strengths of the polyclinic model, would be retained in the provision of specialty care in hospitals rather than primary care.

## Supplementary Information

**Additional file 1.**

**Additional file 2.**

## Data Availability

The datasets used and analyzed during the current study are available from the corresponding author on reasonable request.
