# Supplementary material for: What next for the polyclinic? New models of primary health care are required in many former Soviet Union countries
Source: BMC Prim Care. 2022 Aug 4;23:194. doi: 10.1186/s12875-022-01812-w (PMC9354434; doi:10.1186/s12875-022-01812-w)
Supplement: Supplementary file 2 — Additional file 2. [file 12875_2022_1812_MOESM2_ESM.docx]

**Appendix 2**

Q**uestionnaire of a physician survey on teamwork and coordination of providers in primary health care settings (mostly multi-specialty polyclinics) in Russia (2316 respondents)**

1. Your region of residence? ____________

2. Your gender ____________

– male

– female

3. Your year of birth. __________

4. What position do you hold?

– District therapists

– GP

– Other (please enter it manually)

5. Your polyclinic is located:

– in the administrative centre of your region

– in other city of your region

– in rural area

6. Number of patients in your catchment area (please enter it manually) ____

7. How often does your polyclinic receive information about hospital admissions of patients in the catchment area?

-does not receive at all

-receives in less than 10% of admission cases

-receives in 11-30% of admission cases

-receives in 31-50% of admission cases

-receives in more than 50% of admission cases

-receives in all admission cases

- don’t know

8. How often does district physician in your polyclinic receive information about emergency visits of patients in the catchment area?

-always

-sometimes

-seldom

-does not receive at all

- don’t know

9. Does your polyclinic have a regular practice of visiting patients within first days after their hospital admission with a stroke or a myocardial infarction?

-existed and exists now

-existed and practically does not exist now

-does not exist

-don’t know

10. Is joint development of patients management plans by district physicians and specialists practiced in your polyclinic?

-always

-sometimes

-seldom

-never practiced

- don’t know

11. Is joint involvement of district physicians and specialists in chronic patients management schools practiced in your polyclinic?

-always

-sometimes

-seldom

-never practiced

- don’t know

12. How often, according to your estimate, polyclinics physicians discuss their clinical activity prior to elective admissions with inpatient physicians?

-always

-sometimes

-seldom

-never practiced

- don’t know

13. Is consulting physicians by specialists from diagnostic units on the questions of interpretation of diagnostic tests practiced in your organization?

-always

-sometimes

-seldom

-never practiced

- don’t know

14. What is your estimate of the share of primary district physician visits that are finished with a referral to specialists?

-less than 25%

-26-50%

-51% and more

-don’t know

15. What is your estimate of the share of direct visits to specialists by-passing district

physicians in the overall number of visits to specialists?,

-less than 25%

-26-50%

-51% and more

-don’t know

16. Is there an emergency unit in your polyclinic. If available, is the information on emergency visits transmitted to district physicians?

-always transmitted

-sometimes

-seldom

-never

- don’t know

17. Do district physicians and outpatient specialists contact hospital doctors on managing patients after hospital discharge?

-always

-sometimes

-seldom

-never

- don’t know

18. Do you have training sessions for district physicians with the involvement of specialists in your polyclinic?

-often

-seldom

-never

-don’t know

19. Do you have medical case conferences in your polyclinic?

-often

-seldom

-never

-don’t know

20. How often does your polyclinic receive information on rehabilitation of your patients?

-always

-sometimes

-seldom

-never

- don’t know

21. Do you have an access to electronic medical records on tests, consultations, hospital admissions, other medical services provided in your and other medical facilities of your region?

-always

-sometimes

-seldom

-never

- don’t know

22. Do you refer patients to private medical organizations?

-always

-sometimes

-seldom

-never

- don’t know

23. Do you have feedback from private medical organizations on the results of tests and consultations?

-always

-sometimes

-seldom

-never

- don’t know

24. How do you estimate district physicians’ awareness of a current health status of chronic patients enrolled with them?

-high

-low

-don’t know

25. Do you contact social care workers when your patients need their support?

-always

-sometimes

-seldom

-never

- don’t know
